# Netupitant Inhibits the Proliferation of Breast Cancer Cells by Targeting AGK

**DOI:** 10.3390/cancers16223807

**Published:** 2024-11-12

**Authors:** Zhibo Zhang, Yongzhuang Liu, Hai Wu, Yan Yuan, Zhengrui Liu, Muhammad Sulaiman, Shengtao Yuan, Mei Yang

**Affiliations:** New Drug Screening and Pharmacodynamics Evaluation Center, National Key Laboratory for Multi-Target Natural Drugs, China Pharmaceutical University, Nanjing 210009, China; 3121070232@stu.cpu.edu.cn (Z.Z.); liuyongzhuang97@163.com (Y.L.); wuhai_cpu@163.com (H.W.); drsulaiman66@stu.cpu.edu.cn (M.S.)

**Keywords:** acylglycerol kinase, breast cancer, netupitant, apoptosis, PI3K/AKT/mTOR pathway

## Abstract

**Background**: Currently, there is a significant lack of effective pharmacological agents for the treatment of breast cancer. Acylglycerol Kinase (AGK), a lipid kinase, has been found to be aberrantly expressed in breast cancer and is closely associated with tumor proliferation, migration, and invasion. However, no clinical anti-tumor drugs specifically targeting this kinase have been developed. **Methods**: siRNA was utilized to knock down the AGK gene; CCK8 and colony formation assays were employed to evaluate the in vitro proliferative capacity of tumor cells. Molecular dynamics simulations and BIL assays were conducted to analyze drug binding affinity. Annexin V/PI staining was used to assess apoptotic phenomena; subcutaneous xenograft tumor experiments in nude mice were performed to confirm the in vivo anti-tumor efficacy of the drug. **Results**: Netupitant exhibited stable binding affinity for AGK and interacted with amino acids within the ATP-binding region of the enzyme. The IC_50_ values for the SK-BR-3 and MDA-MB-231 cell lines were determined as 16.15 ± 4.25 µmol/L and 24.02 ± 4.19 µmol/L, respectively; at a concentration of 2.5 µmol/L, Netupitant effectively inhibited clonogenic capacity in breast cancer cells; furthermore, treatment with 10 µmol/L significantly induced apoptosis in these cells. Doses of 50 mg/kg and 100 mg/kg Netupitant markedly suppressed growth rates of subcutaneous xenograft tumors in nude mice while also promoting apoptotic processes. Both in vivo and in vitro studies indicated that Netupitant could inhibit the activation of the PI3K/AKT/mTOR signaling pathway. **Conclusions**: By targeting AGK, Netupitant inhibits its kinase activity, which leads to reduced phosphorylation levels of PTEN, thereby suppressing the activation of the PI3K/AKT/mTOR signaling pathway and ultimately resulting in apoptosis in breast cancer cells.

## Simple Summary

Based on the predicament of the scarcity of clinical drugs for breast cancer and the potential of targeting AGK in the treatment of breast cancer, this study employed a structure-based drug screening strategy that integrates in vitro cell lines and subcutaneous tumor models in nude mice to identify potential marketed drugs targeting AGK, with the aim of providing survival benefits for breast cancer patients.

## 1. Introduction

Breast cancer ranks among the three most prevalent cancers globally [1,2]. Among its various subtypes, triple-negative breast cancer is distinguished by the absence of estrogen receptors (ERs), progesterone receptors (PRs), and HER-2 expression [3]. This subtype is associated with a more aggressive phenotype and a poorer prognosis. Unfortunately, effective therapeutic agents for breast cancer remain limited [4]. In recent years, kinase inhibitors have gained prominence in research, leading to the identification of numerous anti-tumor drugs; notable examples include imatinib [5], which inhibits Abelson (ABL) tyrosine kinase, and the highly selective EGFR inhibitor osimertinib [6]. Data from clinical trials involving kinase inhibitors indicate that only 30% of human kinases are currently under investigation, suggesting significant potential for further development of these agents [7].

AGK (acylglycerol kinase) is a mitochondrial lipid kinase that plays a crucial role in maintaining normal lipid metabolism within mitochondria [8]. It is also closely associated with the transport of mitochondrial proteins, glucose oxidation–reduction reactions, and platelet production [9]. Since the initial discovery by Meryem Bektas et al. indicating that AGK influences the growth and migration of prostate cancer cells [10], an increasing number of studies have demonstrated its pivotal role in the development of various tumors [11]. On one hand, AGK promotes angiogenesis and inhibits apoptosis in liver cancer cells through the activation of NF-κB [12]; on the other hand, it activates the PI3K/AKT/GSK3β signaling pathway, facilitating tumor onset, proliferation, and metastasis in renal cancer [13]. In breast cancer, AGK enhances tumorigenesis and cellular proliferation by inhibiting the transcription factor FOXO1 [14]. Furthermore, AGK modulates glycolysis in CD8+ T cells to influence tumor immunity [15]. Although the critical role of AGK in tumors is increasingly recognized, drug development targeting this protein remains underexplored. Therefore, developing targeted compounds against AGK holds significant clinical promise.

The development of new pharmaceuticals is a lengthy and intricate process, with the complexity of in vivo mechanisms often constraining the application of novel small-molecule drugs. Currently, the repurposing of existing medications has emerged as a significant avenue for targeted therapy: metformin reprograms tryptophan metabolism to enhance CD8+ T cell function in colorectal cancer [16]; stiripentol, an anti-epileptic agent, can disrupt cellular DNA repair mechanisms, rendering tumor cells once again sensitive to radiotherapy and chemotherapy [17]. These agents possess well-defined pharmacokinetic profiles and excellent safety records, allowing for rapid clinical benefits to patients. Therefore, investigating whether there are already-approved drugs that target AGK for breast cancer treatment holds substantial value. Consequently, this study employed a structure-based drug screening strategy that integrates in vitro cell lines and subcutaneous tumor models in nude mice to identify potential marketed drugs targeting AGK, with the aim of providing survival benefits for breast cancer patients.

## 2. Materials and Methods

### 2.1. Chemical Compounds

Natamycin (HY-B0133; purity: 99.30%), Lifitegrast (HY-19344; purity: 99.29%), Gliquidone (HY-B1114; purity: 99.52%), Steviolbioside (HY-N2547; purity: 99.58%), and Netupitant (HY-16346; purity: 99.93%) were obtained from MedChemExpress LLC. (Monmouth Junction, NJ, USA).

### 2.2. Cell Culture

In this research, all cell lines were sourced from Procell Life Science & Technology Co., Ltd. The SK-BR-3 and MBA-MB-231 cell lines were cultured in Dulbecco’s Modified Eagle Medium (Gibco, Waltham, MA, USA), which was enriched with 10% fetal bovine serum (Gibco). The cultures were kept in a 37 °C incubator under a 5% CO_2_ atmosphere (Thermo Fisher Scientific, Waltham, MA, USA).

### 2.3. siRNA Transient Transfection

Cells that were in the exponential growth phase were collected and plated into 6-well plates at a density of 200,000 cells per well. After a 24 h incubation, each well was treated with a mixture consisting of 5 µL of siRNA designed to target AGK (RiboBio, Guangzhou, China) and 5 µL of RNAiMAX (Invitrogen, Carlsbad, CA, USA). The cells were then incubated for an additional 48 h in a 37 °C environment with 5% CO_2_ before proceeding to further analysis.

### 2.4. qRT-PCR

Total RNA was extracted from the cell lines utilizing the RNA-Quick Purification Kit (Yishan, Shanghai, China). About 1 mg of this RNA underwent reverse transcription to generate complementary DNA (cDNA) using HiScript III RT SuperMix (Vazyme, Nanjing, China). Quantitative real-time PCR (qRT-PCR) was conducted with AceQ Universal SYBR qPCR Master Mix (Vazyme), employing GAPDH as an internal control. The primer sequences for AGK included forward primer ‘AC-GAACAGATGAGGCTACC’ and reverse primer ‘AGGG-TATGACTCAAACTACTGG’.

### 2.5. Cell Counting Kit-8 Assay

To evaluate growth curves following siRNA treatment, cells were seeded in 96-well plates at a density of 3000 cells per well. CCK8 solution (Dojindo, Kumamoto, Japan; 10 mL/well) was added at the time points of 24, 48, 96, and 120 h. The mixture was then incubated for 2 h at 37 °C, and absorbance was measured at a wavelength of 450 nm using a Thermo Fisher Scientific spectrophotometer (Waltham, MA, USA). For the IC50 assay, cells in the exponential growth phase were collected and plated into 96-well plates with a density of 3000 cells per well. After an incubation period of 24 h, various concentrations of the drug were applied. Following a treatment duration of 48 h with the drugs, CCK8 solution was introduced to each well. The mixture underwent another incubation for two hours at 37 °C before measuring absorbance at a wavelength of 450 nm (Thermo Fisher Scientific, Waltham, MA, USA).

### 2.6. Virtual Screening and Molecular Dynamics Simulations

The crystal structure of AGK was obtained from the PDB database (PDB ID: 7GCP). The binding region of ATP was selected as the small-molecule targeting region. Smina (v2019-10-15) was used to perform the molecular docking of 2000 FDA-approved drugs [18]. After the docking was completed, the binding information was re-scored by Gnina (v1.0.1) [19]. The specific operation was reported in *Nature Protocol* [20]. Molecular dynamics simulations were performed using Gromacs (v2023.6) [21]. The specific operation was completed according to ‘Protein-ligand’ in GROMACS Tutorials (http://www.mdtutorials.com/gmx/complex/index.html, accessed on 23 June 2023).

### 2.7. BIL Assay

The AGK protein with His tag (LS-G25699-50, LifeSpan BioSciences, Seattle, WA, USA) was specifically captured by the NTA chip (Sartorius, Gottingen, Germany). Once the signal reached 4.5 nm, it was combined with Netupitant at various concentrations. The AGK protein was diluted to 10 µg/mL, with a total volume of 400 µL and solidified for 600 s. Netupitant was diluted with PBST + 5% DMSO to 1 mM. The detection was conducted using the Octet^®^ R8 Non-labeled Protein Analysis System (Sartorius). The detection process was carried out following the steps of Baseline (60 s), Association (45 s), and Dissociation (45 s), and repeated once.

### 2.8. Western Blotting

Following 24 h of drug treatment, the cells were collected and total protein was extracted. Western blotting was conducted according to standard operating procedures. The AGK antibody (PA5-28566) was sourced from Thermo Fisher Scientific (Waltham, MA, USA). Antibodies for Caspase-3 (19677-1-AP), Caspase-9 (10380-1-AP), PARP (13371-1-AP), PTEN (60300-1-Ig), Phospho-PTEN (29246-1-AP), AKT (10176-2-AP), Phospho-AKT (28731-1-AP), mTOR (28273–1–AP), and Phospho-mTOR (28879–1–AP) were obtained from Proteintec (Chicago, IL, USA).

### 2.9. Apoptosis Analysis

The cells were seeded in a 6-well plate at a density of 200,000 cells per well. Following a 12 h incubation, various concentrations of the drug were applied. After 48 h of treatment with the drugs, the cells were collected. The Annexin V-FITC/PI Apoptosis Detection Kit (Vazyme) was utilized to assess programmed cell death. All procedures were performed according to the manufacturer’s instructions.

### 2.10. Colony Formation

The cells were seeded in a 6-well plate at a density of 2000 cells per well. Following a 24 h incubation period, various concentrations of the drug were added. After colony formation, the wells were fixed with 4% paraformaldehyde for two hours, rinsed with phosphate-buffered saline, and subsequently stained with crystal violet for a duration of 24 h.

### 2.11. Nude Mouse Experiment

MDA-MB-231 cells in the exponential growth phase were harvested and resuspended in saline to a concentration of 1 million cells per 100 µL. Subsequently, the diluted cell suspension was injected subcutaneously into the axillary region of nude mice. Once the average subcutaneous tumor volume reached 100 mm^3^, the mice were divided into five groups: control group, 25 mg/kg Netupitant, 50 mg/kg Netupitant, 100 mg/kg Netupitant, and 15 mg/kg paclitaxel group. Each group consisted of five mice; both the control and Netupitant-treated groups received daily oral administration, while the paclitaxel group underwent intravenous injections twice a week. Body weight and tumor volume measurements were taken every two days until the mean tumor volume in the control group reached 1200 mm^3^. Then, the mice were sacrificed for tissue collection. The tumors were photographed and weighted.

### 2.12. Ki-67 and Tunnel Staining

Tumor tissues fixed in 4% paraformaldehyde were subjected to dehydration, embedded in paraffin, and then sectioned. The sections were dried in an oven at 63 degrees Celsius for one hour. Dewaxing was carried out using the LEICAST5020 (Dako, Santa Clara, CA, USA). After antigen retrieval, the sections were incubated overnight at 4 degrees Celsius with Ki-67 antibody (27309-1-AP, Proteintech Group, Inc., Wuhan, China). Blocking, secondary antibody binding, and DAB chromogenic staining were performed using the Autostainer Link 48 (Dako). Following a hematoxylin staining period of one minute, the sections were treated with 0.25% hydrochloric acid alcohol for ten seconds and rinsed with water for five minutes. After sealing with neutral resin, images of the sections were captured. TUNEL staining was conducted according to the protocol provided in the One Step TUNEL Apoptosis Assay Kit (C1086, Beyotime Biotechnology, Haimen, China).

### 2.13. Statistical Analysis

The data are presented as the mean ± standard error of the mean derived from a minimum of three independent experiments. Statistical analyses were performed using SPSS software (Version: 26.0, Abbott Laboratories, Chicago, IL, USA). The significance between different groups was evaluated with Student’s *t*-test. For comparisons involving multiple groups, one-way analysis of variance (ANOVA) followed by Dunnett’s multiple comparison test was applied (* *p* < 0.05; ** *p* < 0.01; and *** *p* < 0.001).

## 3. Results

### 3.1. Netupitant Demonstrates the Potential to Inhibit the Proliferation of Breast Cancer Cells

**Figure 1:**
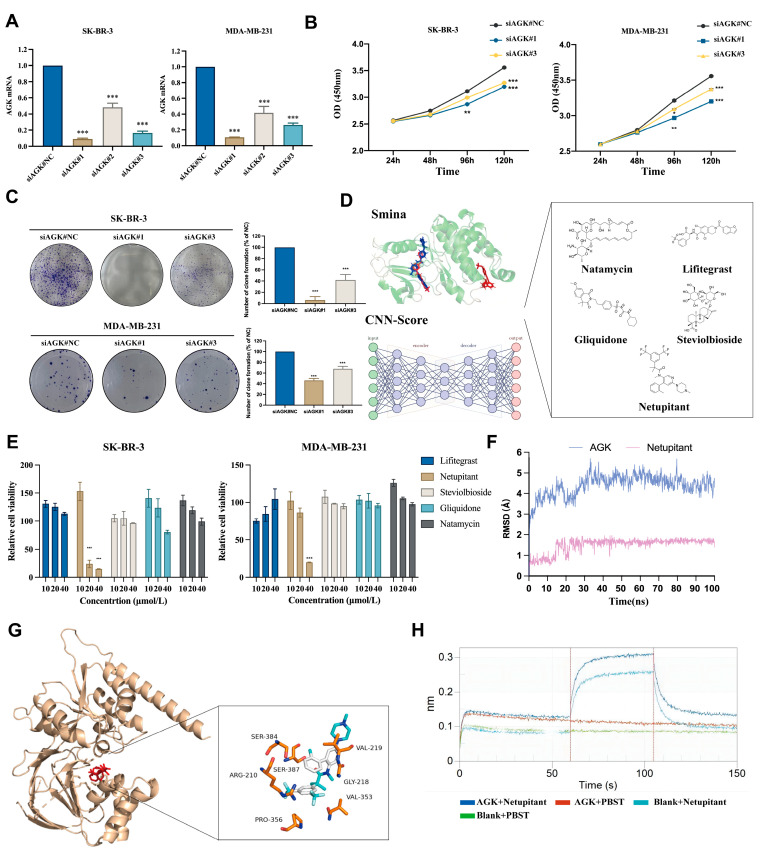
Netupitant demonstrates the potential to inhibit the proliferation of breast cancer cells. (**A**) Validation of gene silencing efficiency for three siRNA sequences. (**B**) Growth curve assay following AGK gene silencing. (**C**) Clonal formation assay subsequent to AGK gene silencing. (**D**) Identification of five candidate compounds targeting AGK through virtual screening. (**E**) Assessment of the inhibitory capacity of these five candidate compounds on breast cancer cell proliferation via CCK8 assay. (**F**) Root Mean Square Deviation (RMSD) analysis of AGK and Netupitant obtained from molecular dynamics simulation. (**G**) Binding site analysis showing where Netupitant interacts with AGK, including the relevant amino acids involved in this interaction. (**H**) BIL analysis conducted to evaluate the affinity between AGK protein and Netupitant. * *p* < 0.05; ** *p* < 0.01; *** *p* < 0.001.

Considering that the role of AGK as a therapeutic target in triple-negative breast cancer has not been definitively established, we employed siRNA to knock down the AGK gene in the SK-BR-3 and MDA-MB-231 cell lines (Figure 1A). The results demonstrated that both the growth capacity (Figure 1B) and clonogenic formation (Figure 1C) of SK-BR-3 and MDA-MB-231 cells were significantly inhibited following AGK knockdown. Subsequently, five FDA-approved drugs were identified as potential candidates for targeting AGK through structure-based virtual screening and neural network scoring (Figure 1D). Among these, Netupitant at a concentration of 40 µmol/L exhibited strong inhibitory effects on both cell lines, suggesting its potential to target AGK and inhibit breast cancer progression, warranting further investigation (Figure 1E). Additionally, molecular dynamics simulations indicated that Netupitant possesses good binding stability with AGK (Figure 1F). The amino acids located at the active site of AGK—SER284, ARG210, PRO356, SER387, VAL219, GLY218, and VAL353—interacted with Netupitant (Figure 1G). Finally, Bio-Layer Interferometry (BLI) assays further validated the interaction between Netupitant and AGK (Figure 1H).

### 3.2. Netupitant Inhibits the Proliferation of Breast Cancer Cells In Vitro by Inducing Apoptosis

**Figure 2:**
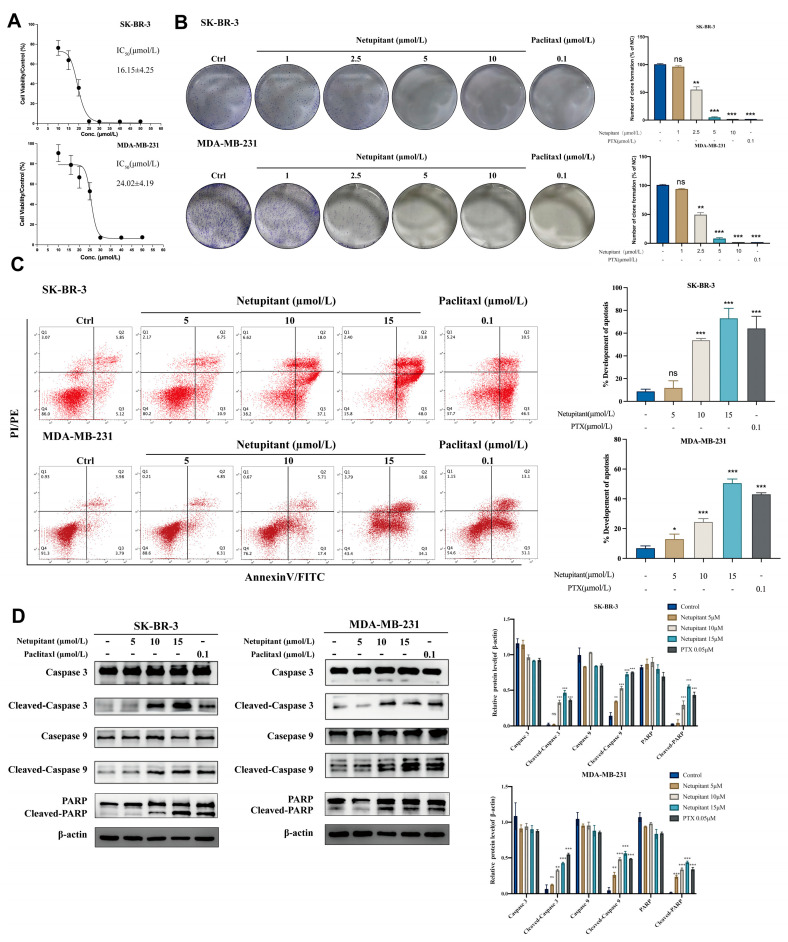
Netupitant inhibits proliferation of breast cancer cells in vitro by inducing apoptosis. (**A**) IC50 values of Netupitant on SK-BR-3 and MDA-MB-231 cell lines. (**B**) Clonal formation assay following treatment with Netupitant. (**C**) Annexin V/PI staining for apoptosis assessment after 48h treatment. (**D**) Upregulation of apoptosis-related proteins subsequent to Netupitant treatment. * *p* < 0.05; ** *p* < 0.01; *** *p* < 0.001; ns: no significant difference.

Subsequently, we investigated the specific concentration of Netupitant that inhibits the proliferation of breast cancer cells. Initially, the CCK-8 assay revealed that the IC50 values of Netupitant in SK-BR-3 and MDA-MB-231 cells were 16.15 ± 4.25 µmol/L and 24.02 ± 4.19 µmol/L, respectively (Figure 2A). Notably, a concentration of 2.5 µmol/L of Netupitant exhibited a significant ability to inhibit tumor colony formation (Figure 2B). In alignment with previous research on the influence of AGK on cell apoptosis [22], silencing the AGK gene using siRNA induced apoptosis in breast cancer cells (Figure S1E), and Annexin V/PI staining confirmed that concentrations of 10 µmol/L and 15 µmol/L of Netupitant induced apoptosis in breast cancer cells in a dose-dependent manner (Figure 2C). Furthermore, the upregulation of Cleaved-Caspase3, Cleaved-Caspase9, and Cleaved-PARP proteins following exposure to Netupitant further substantiated its apoptotic effects (Figure 2D).

### 3.3. Netupitant Inhibits the PI3K/AKT/mTOR Signaling Pathway In Vitro

**Figure 3:**
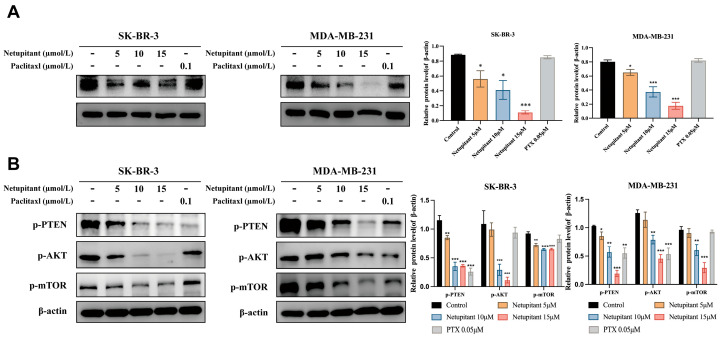
Netupitant inhibits PI3K/AKT/mTOR signaling pathway in vitro. (**A**) Expression levels of AGK protein following treatment with Netupitant. (**B**) Assessment of phosphorylation status of proteins associated with PI3K/AKT signaling pathway after Netupitant treatment. * *p* < 0.05; ** *p* < 0.01; *** *p* < 0.001.

Following the confirmation of targetability and in vitro anti-tumor effects, this study further explored the molecular mechanisms underlying Netupitant. Given that AGK has the capacity to phosphorylate and inactivate PTEN, the PI3K/AKT/mTOR signaling pathway became a primary focus of our investigation. Western blot analysis demonstrated that treatment with Netupitant at concentrations of 10 µmol/L and 15 µmol/L significantly downregulated AGK protein expression (Figure 3A) in both cell lines while markedly reducing the phosphorylation levels of PTEN, AKT, and mTOR (Figure 3B). These findings confirm that Netupitant modulates the PI3K/AKT/mTOR signaling pathway by targeting AGK.

### 3.4. Netupitant Inhibits the Proliferation of Breast Cancer Cells In Vivo

**Figure 4:**
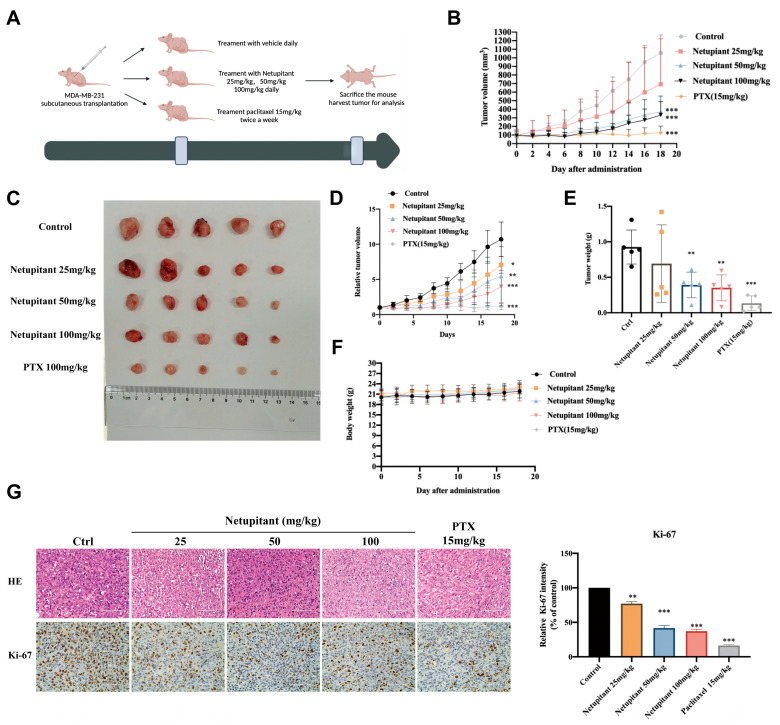
Netupitant inhibits proliferation of breast cancer cells in vivo. (**A**) Overview of in vivo experimental procedure. (**B**) Tumor volume growth curve. (**C**) Photograph of excised tumor specimens. (**D**) Relative tumor volume change curve. (**E**) Measurement of tumor weight. (**F**) Body weight progression curve. (**G**) HE and Ki-67 staining images of tumor tissues. * *p* < 0.05; ** *p* < 0.01; *** *p* < 0.001.

As Netupitant is an FDA-approved drug, its bioavailability has been established; therefore, in vitro efficacy should correlate with in vivo efficacy. In this study, a subcutaneous tumor model was developed using MDA-MB-231 cells implanted in nude mice and treated with Netupitant at doses of 25 mg/kg, 50 mg/kg, and 100 mg/kg. As illustrated in Figure 4B–E, both the 50 mg/kg and 100 mg/kg doses of Netupitant significantly inhibited tumor growth, demonstrating therapeutic effects on various parameters including tumor volume, relative tumor volume, and tumor weight. However, no significant difference in therapeutic efficacy was observed between the 50 mg/kg and 100 mg/kg doses of Netupitant. Furthermore, none of the concentrations of Netupitant affected the body weight of the mice, indicating a high level of safety (Figure 4F). Ki-67 staining further confirmed the reduction in the rate of tumor proliferation following treatment with Netupitant (Figure 4G).

### 3.5. Netupitant Induces Apoptosis in Tumor Cells In Vivo via the PI3K/AKT/mTOR Signaling Pathway

**Figure 5:**
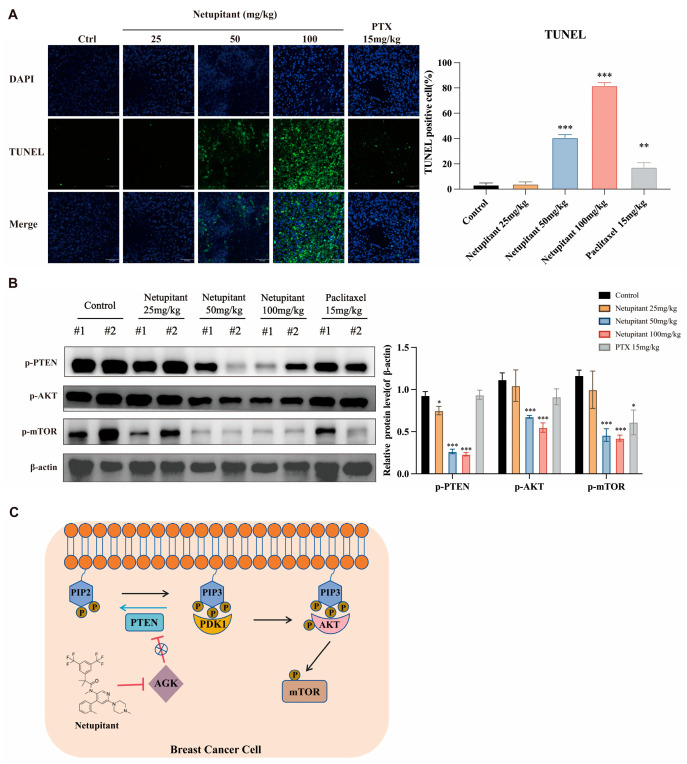
Netupitant induces apoptosis in tumor cells in vivo through the PI3K/AKT/mTOR signaling pathway. (**A**) TUNEL staining images of tumor tissues. (**B**) The degree of phosphorylation of PI3K/AKT signaling pathway-related proteins in tumor tissues following Netupitant treatment. #1: mice1; #2: mice2; * *p* < 0.05; ** *p* < 0.01; *** *p* < 0.001. (**C**) Netupitant, by inhibiting AGK, decreases the phosphorylation of PTEN, and thereby reduces the phosphorylation activation of AKT and mTOR.

Finally, this study conducted TUNEL staining on subcutaneous tumor tissues. In the groups treated with Netupitant at doses of 50 mg/kg and 100 mg/kg, a significant increase in fluorescence intensity was observed in tumor tissue sections, indicating a higher presence of fragmented DNA and reflecting a pronounced apoptotic effect (Figure 5A). Consistent with findings from in vitro experiments, the administration of Netupitant at both 50 mg/kg and 100 mg/kg significantly reduced the phosphorylation levels of PETN, AKT, and mTOR (Figure 5B). Consequently, it is evident that Netupitant mediates its anti-proliferative effects on breast cancer cells by targeting AGK and inhibiting the PI3K/AKT/mTOR signaling pathway.

## 4. Discussion

Netupitant was approved by the U.S. Food and Drug Administration (FDA) in October 2014 for use in conjunction with palonosetron to prevent both acute and delayed nausea and vomiting, particularly those associated with highly emetogenic chemotherapy [23]. As a potent neurokinin-1 receptor antagonist, Netupitant inhibits the interaction between substance P and the NK1 receptor, thereby disrupting or delaying the transmission of emetic signals [24]. Its pharmacokinetic profile is favorable: blood concentrations can be measured within 15 min to 3 h following oral administration, with maximum concentration (C_max_) achieved within 4–5 h at approximately 400–500 ng/mL [25]. Furthermore, Netupitant exhibits a large volume of distribution (VZ), with an average VZ of 1982 L observed after a single oral dose of Netupitant/palonosetron in cancer patients, indicating extensive systemic distribution [23,26]. Currently, there are no reports documenting anti-tumor activity for Netupitant; however, this study reveals its potential anti-tumor activity, which has certain clinical implications.

The treatment of triple-negative breast cancer poses significant challenges due to the lack of classification antigens on the surface of cancer cells, which renders this subtype resistant to hormone therapy and targeted HER2 therapies [27]. As a result, clinical management often relies on cytotoxic chemotherapy agents such as paclitaxel and cisplatin, leading to considerable side effects that substantially reduce patient adherence to treatment [28]. Therefore, the development of molecular targeted therapies specifically aimed at addressing triple-negative breast cancer holds substantial promise. Xi Wang et al. [14]. identified an abnormal expression of AGK in breast cancer and demonstrated that the knockdown of AGK in HER2-positive breast cancer cells could inhibit their proliferation and migration. However, this study did not assess the effects of targeting AGK in triple-negative breast cancer. In light of this gap, our research incorporated the MDA-MB-231 cell line into the experimental design. The results indicated that AGK gene expression is critical for both subtypes of breast cancer cells, underscoring its potential as a broad-spectrum therapeutic target for breast cancer treatment.

Considering that the catalytic substrate of kinases requires ATP binding [29], we employed a competitive binding strategy by utilizing the ATP-binding domain of AGK as the active site for virtual screening. Molecular dynamics simulations and BLI assays confirmed the interaction between the compound Netupitant and AGK, while key amino acids involved in this interaction site were preliminarily identified, establishing a foundation for potential future drug development. The findings from the initial screening and IC_50_ analysis indicate that Netupitant exhibits a more pronounced inhibitory effect on SK-BR-3 cell proliferation compared to MDA-MB-231. This discrepancy may be attributed to varying expression levels of AGK protein in these two cell lines (Figure S1B) or could be associated with the heightened activation of the PI3K/AKT/mTOR signaling pathway in triple-negative breast cancer [30,31]. Notably, clonogenic assays reveal that Netupitant exerts a robust inhibitory effect on both cell lines at a concentration of 2.5 µmol/L. Netupitant showed a significantly weaker inhibition of proliferation in normal breast cells MCF-10A than in SK-BR-3 cells, suggesting that it may not have significant cytotoxic effects. Clonogenic assays assess both cellular proliferation and stemness; tumor cell stemness is linked to tumorigenesis and recurrence. Furthermore, AGK protein has been implicated in promoting tumorigenesis [32]. These results suggest that Netupitant not only inhibits cellular proliferation but may also play a role in preventing tumor recurrence.

AGK, a lipid kinase, catalyzes the conversion of monoacylglycerol and polyacylglycerol into phosphatidic acid (PA) and lysophosphatidic acid (LPA) within mitochondria [33]. Recent studies have demonstrated that AGK exhibits typical characteristics of traditional protein kinases [11]. The tumor-promoting mechanisms associated with AGK are primarily linked to its protein kinase activities. Notably, AGK has been shown to activate the PI3K/AKT signaling pathway [34], thereby inhibiting cell apoptosis. Specifically, AGK phosphorylates three residues on PTEN (Ser380, Thr382, and Thr383), leading to its inactivation and an increase in AKT phosphorylation levels [15]. On one hand, activated AKT phosphorylates FOXO1, which reduces its nuclear translocation and inhibits apoptotic processes [14]; on the other hand, activated AKT also phosphorylates GSK3β, resulting in decreased β-catenin degradation and promoting its nuclear translocation. This enhances the stemness of nasopharyngeal carcinoma cells—a finding consistent with clonal formation results observed in this study [32]. Furthermore, by combining observations of significant apoptotic effects seen in breast cancer cells exposed to Netupitant with the critical role of mTOR signaling in suppressing breast cancer cell apoptosis [35,36], this study elucidates the relationship between Netupitant, the PI3K/AKT/mTOR pathway, and apoptosis through both in vitro and in vivo experiments. It is worth noting that Phospholipase D (PLD), a lipid-metabolizing enzyme, also regulates the PI3K/AKT/mTOR signaling pathway in cancer cells [37]. It has been demonstrated that inhibiting PLD activity allows metformin to create a synthetic lethal phenotype with rapamycin in breast cancer cells [38]. Future investigations will focus on analyzing downstream effector molecules related to mTOR signaling and the interplay between AGK and PLD/mTOR pathways.

However, this study has certain limitations. Firstly, the role of AGK in the metastasis of breast cancer and the potential of Netupitant to inhibit the migration and invasion of triple-negative breast cancer cells remain unexplored. Secondly, there has been no investigation into whether the suppression of AGK induces mitochondrial-mediated apoptosis. Additionally, a relationship between the NK1R gene and tumors has been reported, so we searched the CCLE database and selected the cell line MD-MBA-231, which has a negligible natural expression of NK1R (Figure S1D). The consistency of molecular signal changes in SK-BR-3 and MDA-MB-231 cells after Netupitant treatment can preliminarily exclude the influence of the NK1R receptor in the experiment. However, whether Netupitant, as an inhibitor of two targets, can achieve better effects in triple-negative breast cancer is also a worthwhile topic for future exploration. Addressing these issues will be a primary focus for future research.

## 5. Conclusions

This study demonstrates that Netupitant effectively inhibits the proliferation of breast cancer cells by targeting AGK. Netupitant inhibits the activation of the PI3K/AKT/mTOR signaling pathway by suppressing the AGK protein, inducing the apoptosis of breast cancer cells. In summary, this study establishes a foundational basis for the repurposing of Netupitant and facilitates future drug development.

## Data Availability

All relevant data are provided within the paper and its Supplementary Materials. The data supporting the findings of this study are available from the corresponding author upon reasonable request.

## Supplementary Materials

The following supporting information can be downloaded at: https://www.mdpi.com/article/10.3390/cancers16223807/s1, Figure S1: (A) The inhibitory effect of X on the proliferation of MCF-10A and SK-BR-3 cells was detected by CCK8 assay. (B) Expression of AGK protein in different cell lines. (C) Determination of the IC50 values of PTX on SK-BR-3 and MDA-MB-231 cells using the CCK8 assay. (D) The mRNA expression of AGK in the Cancer Cell Line Encyclopedia (CCLE) database. (E) Apoptosis assay following AGK gene knockdown via siRNA. Figure S2: Uncropped Western blot images.
