# Supplementary material for: Netupitant Inhibits the Proliferation of Breast Cancer Cells by Targeting AGK
Source: Cancers (Basel). 2024 Nov 12;16(22):3807. doi: 10.3390/cancers16223807 (PMC11592365; doi:10.3390/cancers16223807)
Supplement: Supplementary file 1 [file cancers-16-03807-s001.zip › cancers-3237321-supplementary.pdf]

Supplementary Materials: Netupitant Inhibits the Proliferation of Breast Cancer Cells by Targeting AGK

Zhibo Zhang, YongZhuang Liu, Hai Wu, Yan Yuan, Zhengrui Liu, Muhammad Sulaiman, Shengtao Yuan and Mei Yang

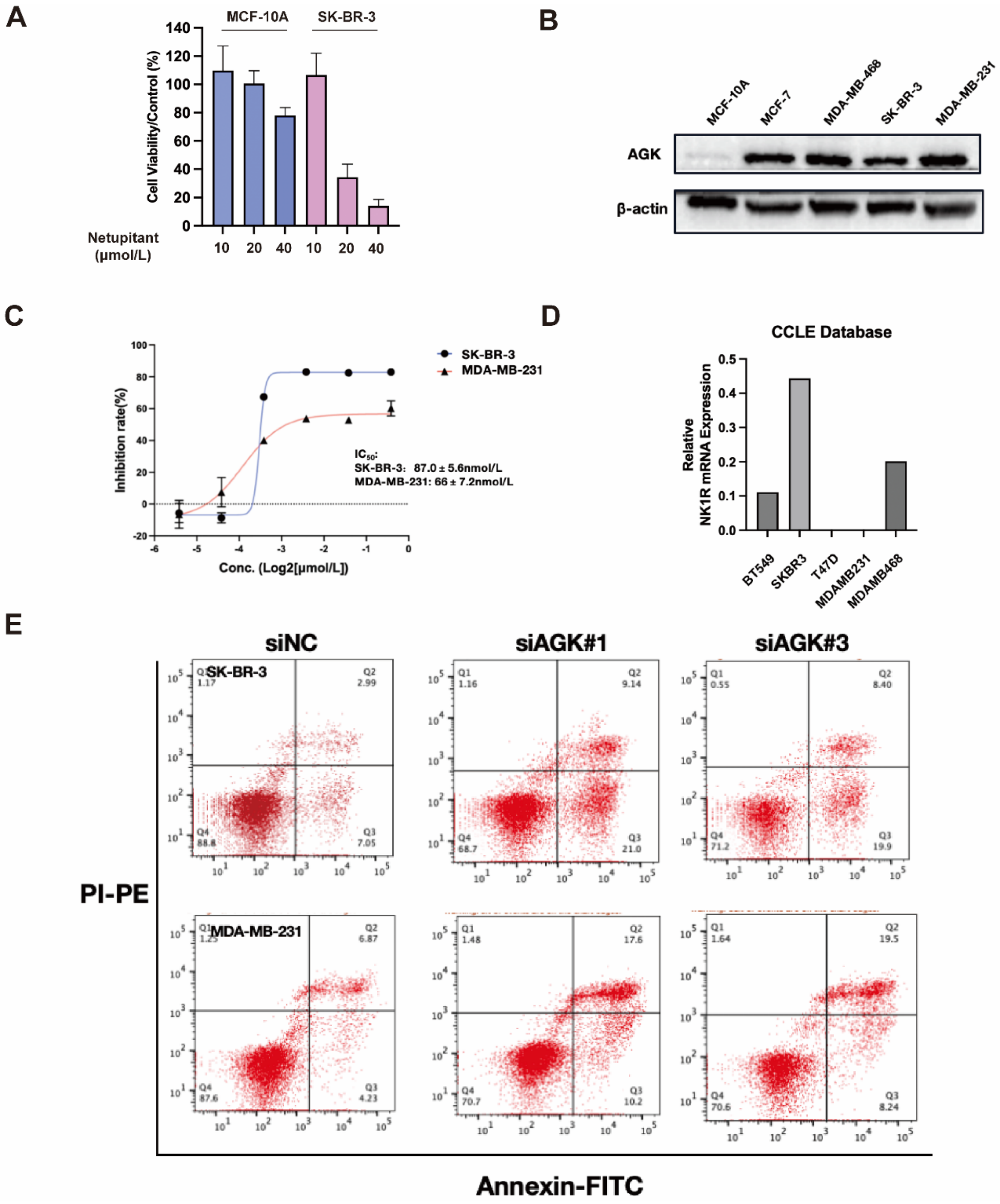

Figure S1. (A) The inhibitory effect of X on the proliferation of MCF-10A and SK-BR-3 cells was detected by CCK8 assay. **(B)** Expression of AGK protein in different cell lines. **(C)** Determine the IC50 values of PTX on SK-BR-3 and MDA-MB-231 cells using the CCK8 assay. **(D)** The mRNA expression of AGK in Cancer Cell Line Encyclopedia (CCLE) database. **(E)** Apoptosis assay following AGK gene knockdown via siRNA.

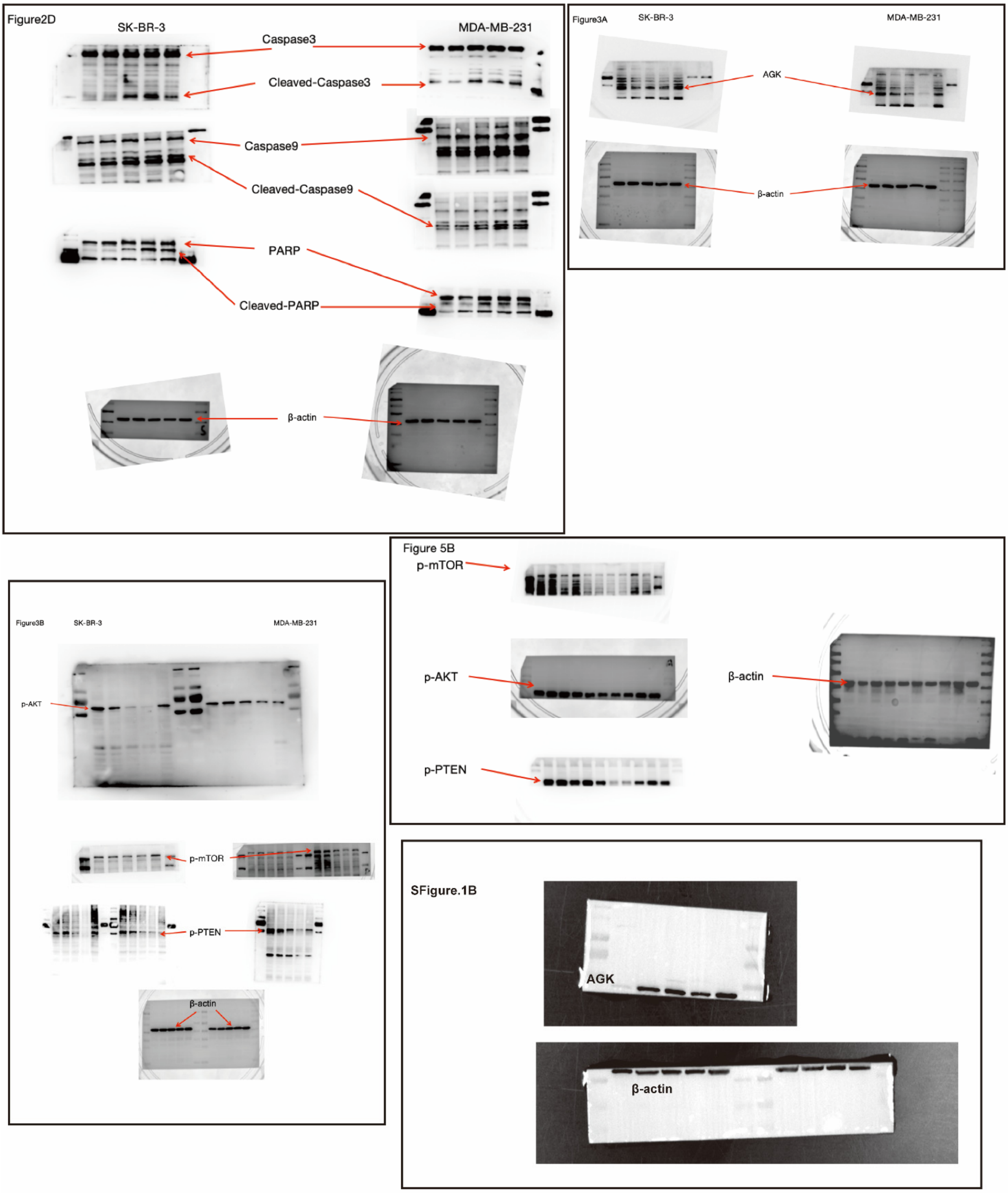

Figure S2. Uncropped Western Blot images.
