# Supplementary material for: Netupitant Inhibits the Proliferation of Breast Cancer Cells by Targeting AGK
Source: Cancers (Basel). 2024 Nov 12;16(22):3807. doi: 10.3390/cancers16223807 (PMC11592365; doi:10.3390/cancers16223807)

Figure2D

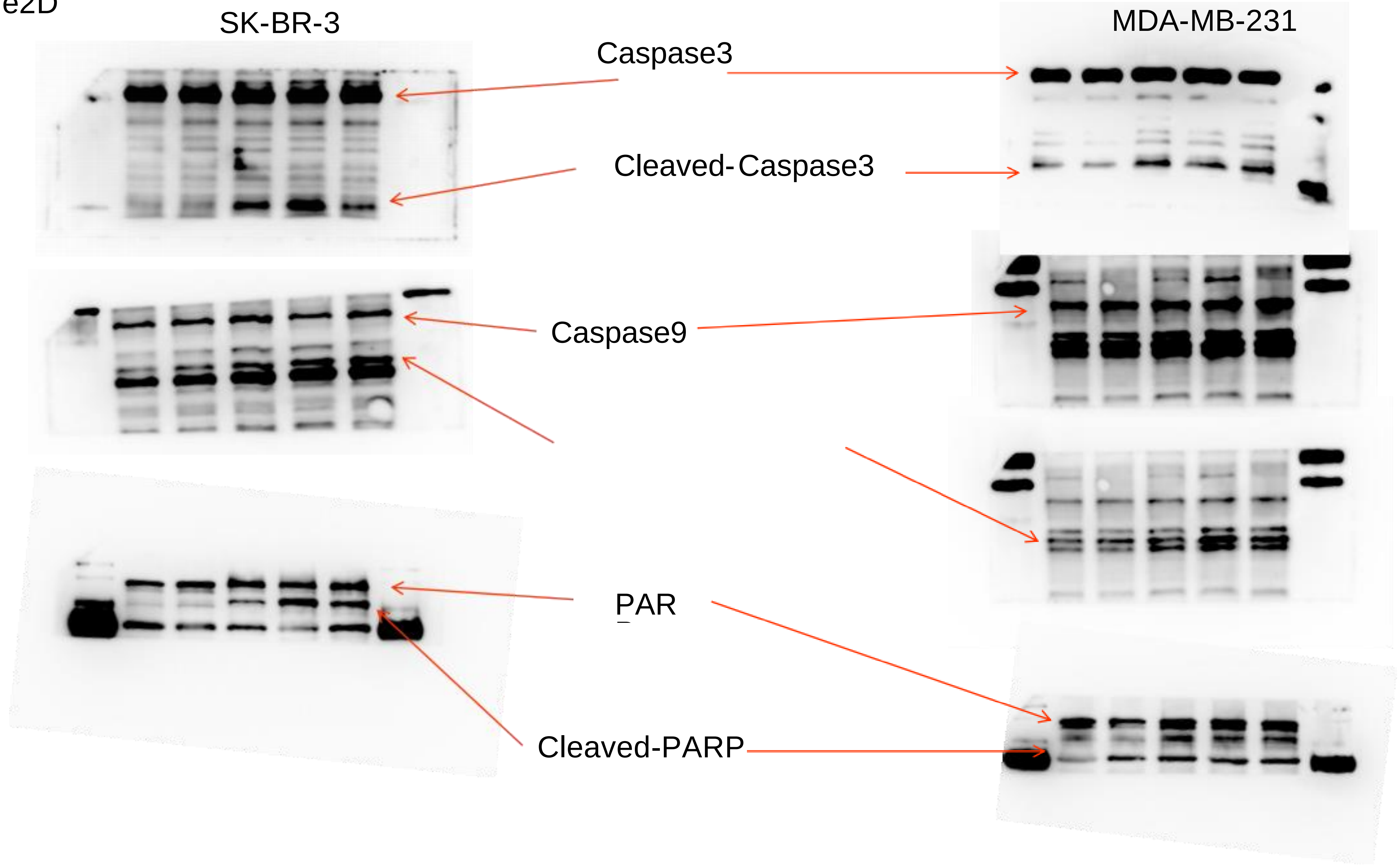

SK-BR-3
MDA-MB-231
Caspase3
Cleaved- Caspase3
Caspase9
PAR
Cleaved-PARP

Figure2D

SK-BR-3 MDA-MB-231

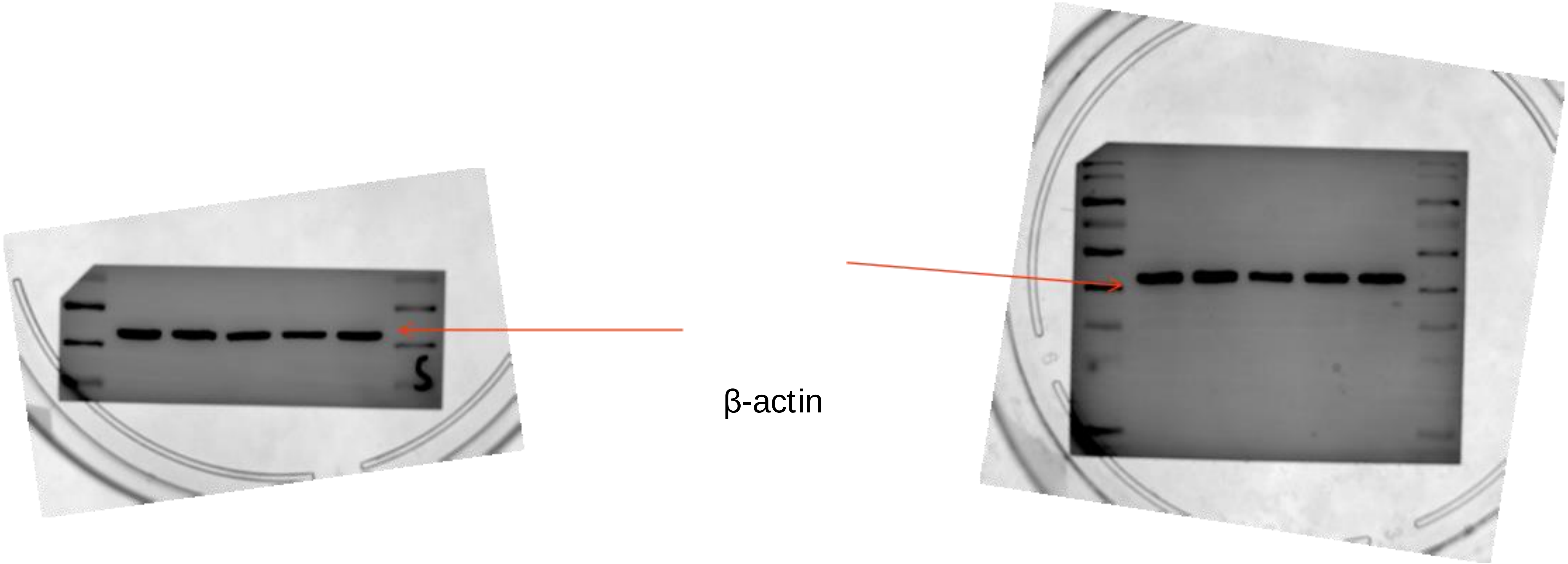

Figure3A

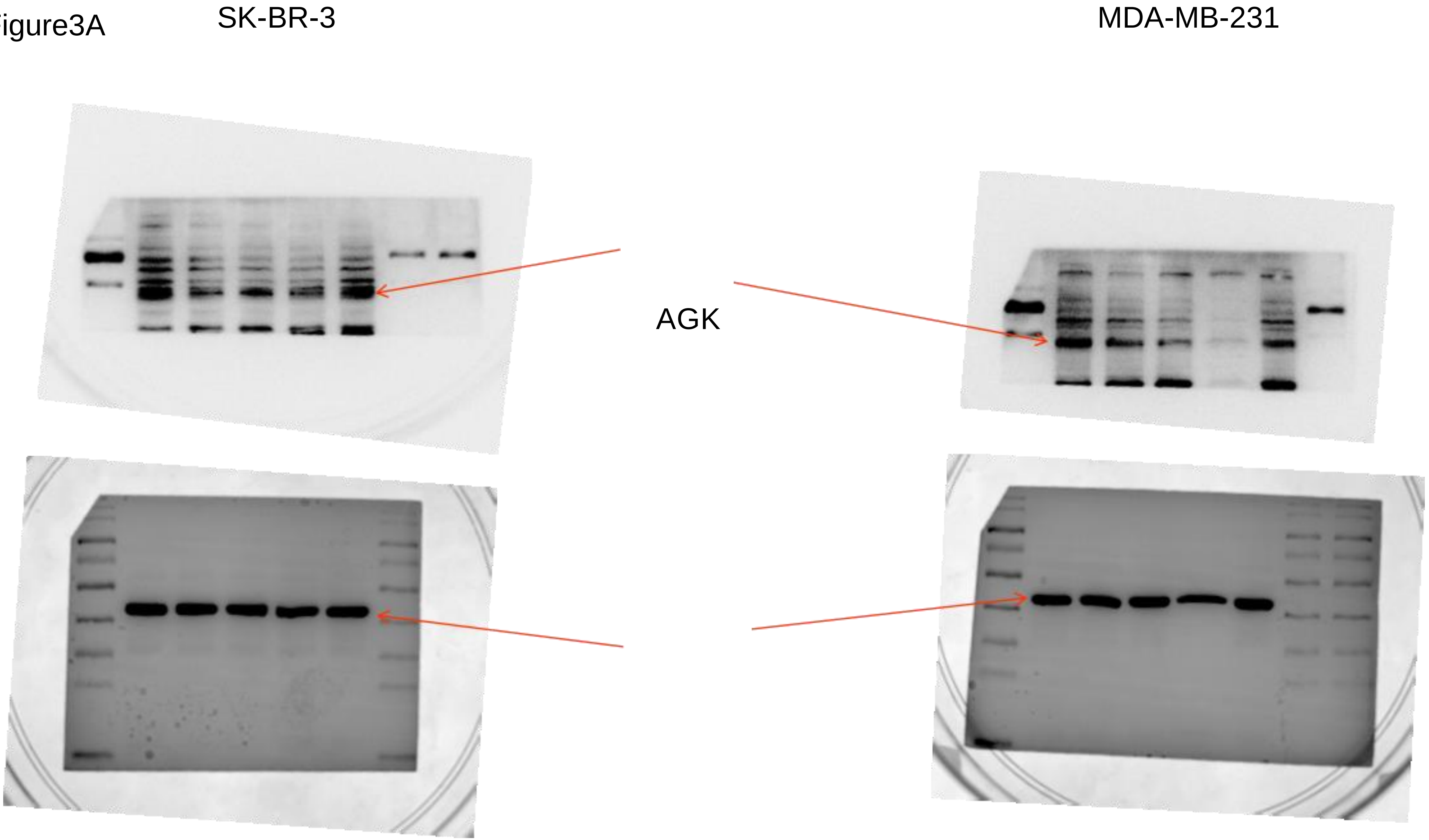

β-actin

Figure3B SK-BR-3 MDA-MB-231

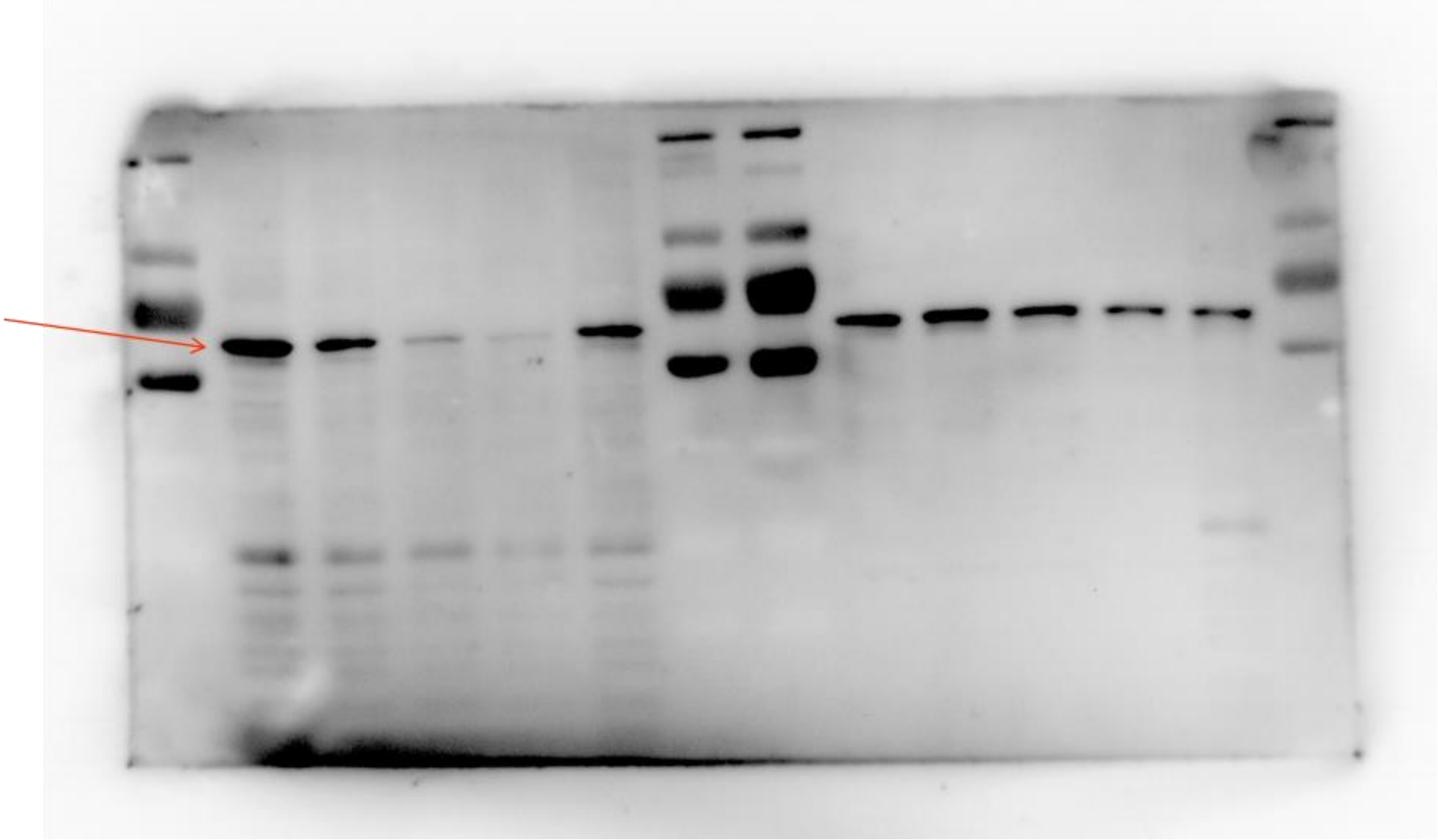

p-AKT

Figure3B

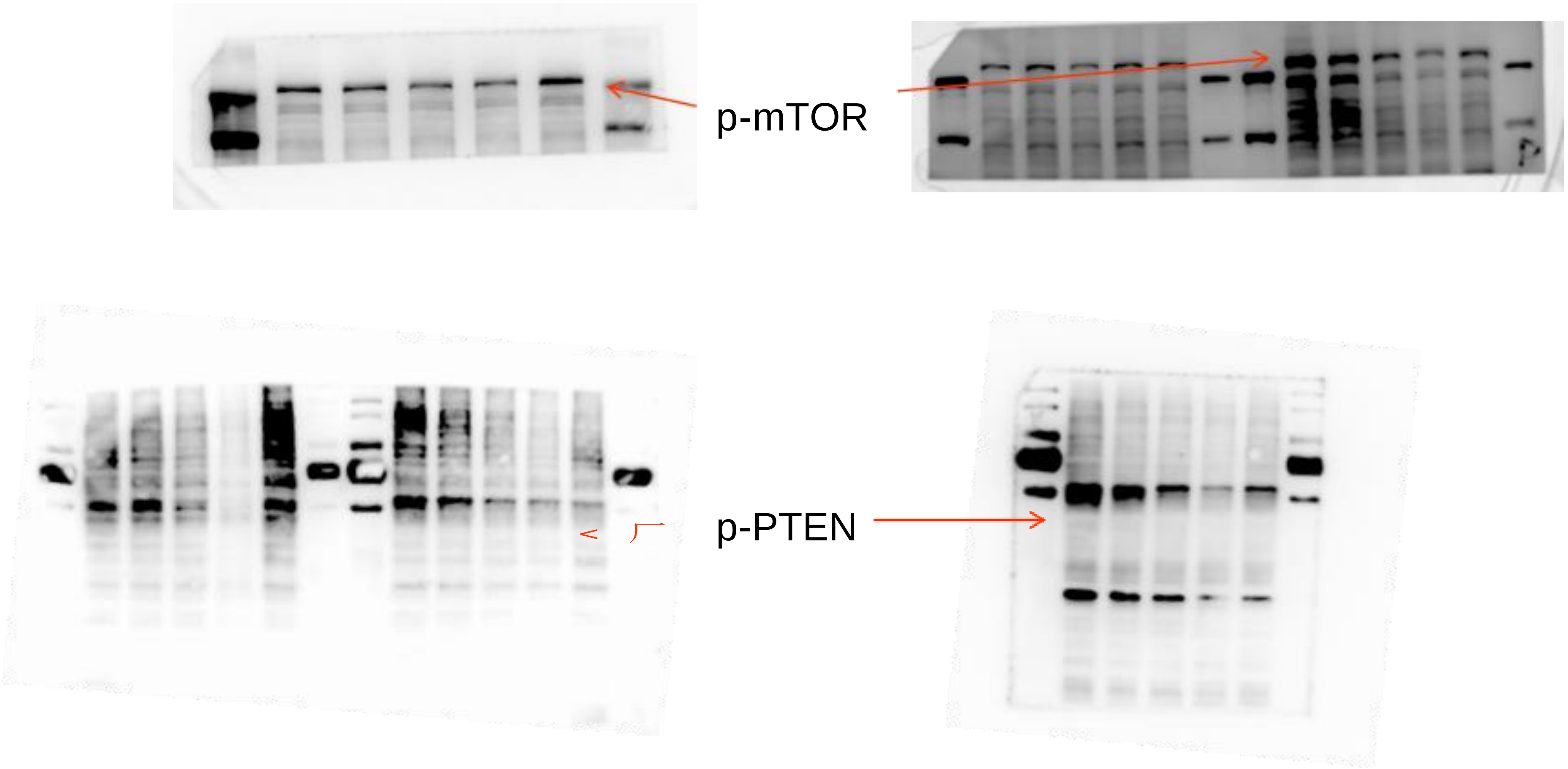

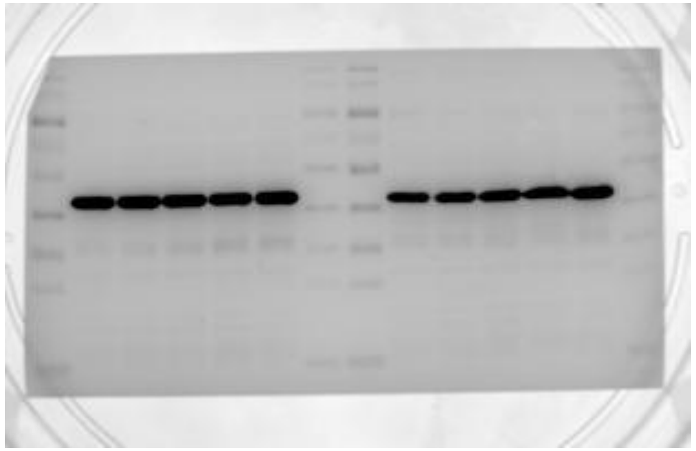

Figure 5B

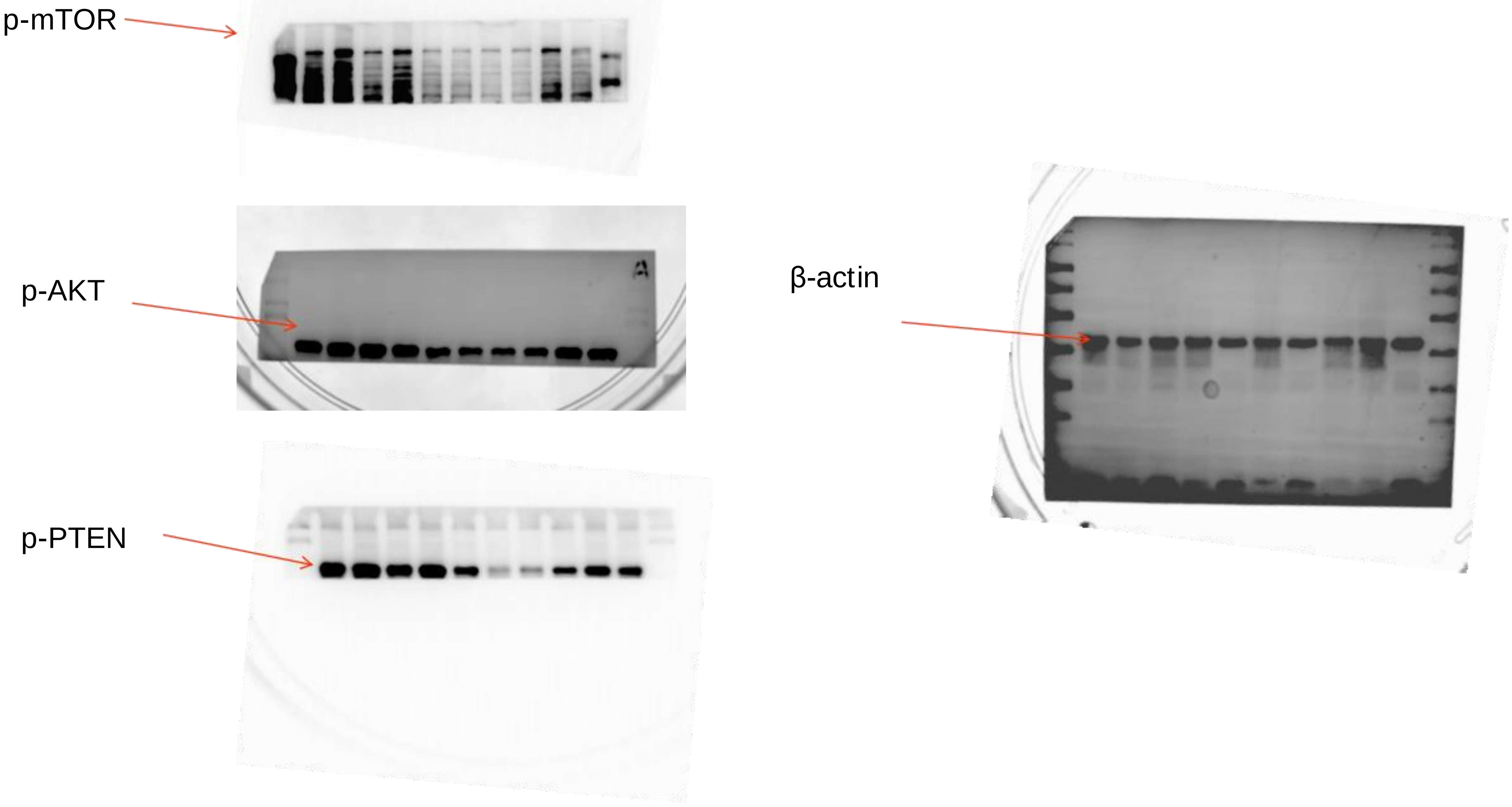

SFigure.1B

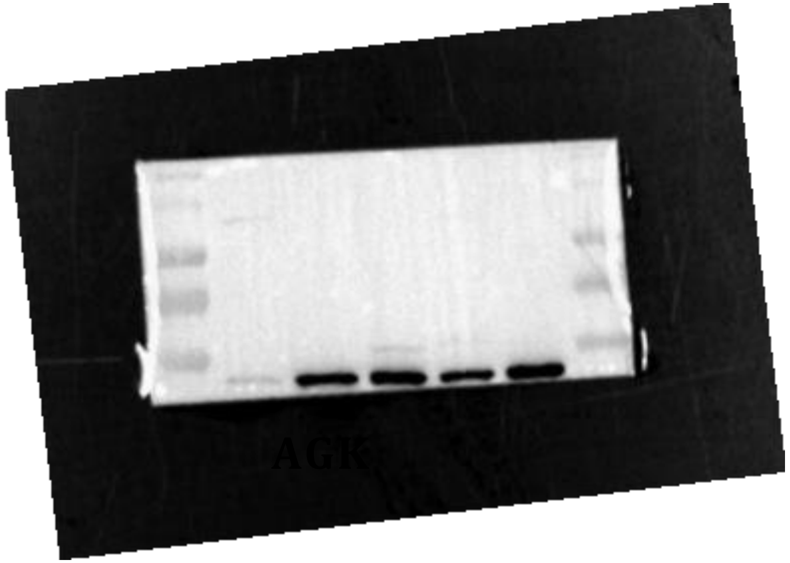

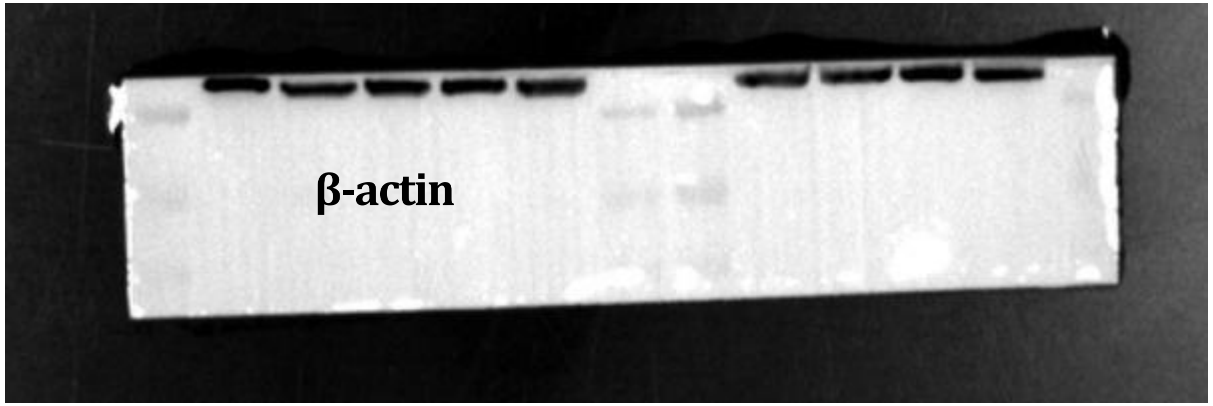

Supplement: Supplementary file 1 [file cancers-16-03807-s001.zip › uncropped Western Blot images.pdf]
